# Parents’ Perspectives on Radicalization: A Qualitative Study

**DOI:** 10.1007/s10826-018-1048-x

**Published:** 2018-03-14

**Authors:** Elga Sikkens, Marion van San, Stijn Sieckelinck, Micha de Winter

**Affiliations:** 1 Department of Pedagogical and Educational Sciences, Faculty of Social and Behavioral Sciences, Utrecht University, PO Box 80140, 3508 TC Utrecht, The Netherlands; 2 Research Institute Risbo, Erasmus University Rotterdam, Rotterdam, The Netherlands; 3 Department of Pedagogical and Educational Sciences, Utrecht University, Utrecht, The Netherlands

**Keywords:** Parenting, Upbringing, Radicalization, Ideology, Adolescent development, Parenting style

## Abstract

Radicalization of young people might be influenced by the way parents react towards the development of political or religious ideals. However, these reactions have hardly been explored. This study aimed to discover how parents reacted to the development of extreme ideals, and why they responded in the way that they did. To gain knowledge about the influence of parents on adolescents who developed extreme ideals, 82 in-depth interviews were held with adolescents and young adults who held extreme ideals. Interviews were also held with the parents or siblings of each adolescent and young adult. In line with parenting style theory, it was found that parents react in four possible ways: (1) by rejecting, (2) applauding, (3) ignoring, or (4) discussing the (extreme) ideals of their children. Few parents discuss ideals and values with their child, and this paper tries to show why (e.g., powerlessness, disassociation, occupation with other problems, believing it to be a phase that will pass, or that their reaction would not help). Most parents struggle to cope with radicalization and do not know how to react. Support and control are potentially important tools for parents to use to combat the development of extreme ideology.

## Introduction

Every so often our societies are confronted with terrorist violence as a result of radicalization: the Paris bombings; Anders Breivik’s attack on Utøya; or the recent flow of young people that join the jihad, led by the Islamic State. Many scholars have searched for motives and causes of radicalization (Borum 2004; Moghaddam 2005; Sageman 2004; Veldhuis and Staun 2009). However, identifying radicalization is difficult, partly because no agreement exists on how to define radicalization. Some scholars define radicalization as a cognitive process; whereas, others consider it to be a process of increased violence (Bartlett et al. 2010; Vidino and Brandon 2012). For the purpose of this discussion we will use the definition of McCauley and Moskalenko (2011). They defined radicalization as, “changes in beliefs, feelings and behavior in the direction of increased support for a political conflict. Radicalization can involve the movement of individuals and groups to legal and nonviolent political action (activism) or to illegal and violent political action (radicalism)” (McCauley and Moskalenko 2011, p. 82). Mandel (2009) noticed that being radical only exists in comparison with others. Considering this relative meaning of radicalization, we would like to add to the definition that radicalization is the process through which an adolescent or young adult develops ideals that are severely at odds with those of their family or the mainstream.

Radicalization is often seen as a single event, but it is possibly more valuable to approach radicalization as a transitional process, influenced by multiple life experiences. These processes are often marked by a sequence of childhood to adulthood transitions, consisting not just of major life events, but also of the more gradual transitions that are common to growing up (Sieckelinck et al. 2017). Transition can be described as the process by which people cope with change, and the reconstruction of a positive identity is then an essential component of this process (Kralik et al. 2006). During adolescence and emerging adulthood, all young people go through developmental stages, entailing transitions in which they, for example, address questions about who they are, about detachment from their parents, and about worldview and religion (Arnett 2014). This development also includes the development of moral principles, guiding decision-making in daily life. During these developmental stages young people may also encounter difficulties, such as financial problems, conflict with a parent, social exclusion, cultural humiliation, feelings of uselessness, etc., which they have to overcome (Sieckelinck et al. 2017). These difficulties could be seen as underlying causes of radicalization; which have been described as “push factors”, pushing adolescents towards radical groups (Schmid 2013). Although they are a necessary condition, push factors do not cause radicalization directly. People are also attracted to radical groups by the positive features and benefits of membership—the so-called “pull factors” (Hassan 2012). For example, various studies have shown that young people who are experiencing uncertainty are attracted to highly-structured, supportive groups with clear boundaries that they can identify with (Hogg 2014; Kotnis 2015).

Although emerging adults try to develop their identity apart from their parents in order to become independent (Arnett 2014), perhaps parents can influence the development of their adolescent so that their transition into adulthood will not lead to radicalization. Smith et al. (2011) found that emerging adults are often deprived of moral formation, because parents and teachers avoid talking about controversial moral issues. They recommend that schools offer classes in basic moral reasoning to help young people address moral issues and challenges. Moreover, classic research on parenting styles has shown that the best child outcomes, in terms of health and social development, are produced by a combination of parental warmth and control (Maccoby and Martin 1983). Both affectionate parent-child interaction and parental discipline possibly have a beneficial effect on children’s moral development (Hoffman 2000; Smetana 1999). In contrast, lack of warmth, lack of support, lack of supervision, and harsh parenting may increase the chances that children will become delinquent (Hoeve et al. 2008). Of course, the parental role changes as children grow older: earlier research has shown that, in late adolescence and early adulthood, support from parents matters more than control and supervision (Johnson et al. 2011). Although young peoples’ independence increases as they grow older, emerging adults remain closely connected to their parents throughout their twenties (Arnett 2014). Emotional attachment to parents remain important and has a positive influence on identity development and the overall well-being of young adults, which also leads to lower rates of delinquency (Johnson et al. 2011).

Although it is possible that lack of support and control leads to deviant behavior, Kerr et al. (2009) found that parents often disengage when their adolescent starts to display problematic behavior. They found that rather than increasing their monitoring of their child when they noticed that he or she was involved in deviant behavior, parents tended to give the child more autonomy (Kerr et al. 2009). Kerr et al.’s (2009) data suggest that parents reduce their monitoring, because they are intimidated by their child’s behavior, or because they are emotionally excluded by the child. This confirmed earlier research suggesting that parents become less supportive and controlling of their children, because they are scared by their aggressive behavior and antisocial identity (Baumrind and Moselle 1985; Stice and Barrera 1995). Thus, it seems plausible that parents would react to radicalization in similar ways.

Becker (2008, pp. 342–348) explored the family dynamics within the radicalization process. He focused on the interaction between young people that endorsed extreme-right ideals and their parents. Becker differentiated between four types of interaction within “rightwing families”: (1) the *protected (“geschützte”)* family, (2) the *threatened (“gefährdete”)* family, (3) the *settled (“eingerichtete”)* family, and (4) the *abandoned (“verlassene”)* family. In a protective (“geschützte”) family, the parents would talk substantively to their children about their ideology, without ever withdrawing support. In a threatened (“gefährdete”) family, the parents and their child talk about politics and ideology, but the communication is mainly unilateral, as the child tries to convert the parent towards his ideals. In Becker’s (2008, pp. 342–348) *settling* (“eingerichtete”) family, the parent agrees with the right-wing ideology of the child to a certain extent and, therefore, does not intervene. Parents and the child do not discuss politics and ideology substantively, but the parents might try to reduce the ideology when right-wing behavior becomes too apparent. In abandoning (“verlassene”) families, political and ideological issues are not discussed; and the parental response can be described as indifferent: parents have trouble controlling the behavior of their children.

Sikkens et al. (2017) focused on the reactions of parents when they were confronted with radicalization. They found that parents’ reactions to extreme ideology often changed as their children became radical. At first, parents were pleased by their child’s new or renewed interest in religion or politics; however, when they noticed their child’s fanaticism, they would reject or ignore his or her beliefs. Also, parents’ response to radicalization was sometimes different from what one would expect from their general parenting style. This is probably because parents do not know how to cope with a child’s endorsement of extreme ideas or the creeping process of radicalization; thus, it seems that there is a degree of parental uncertainty about how to handle the (potential) radicalization of a child (Pels and De Ruyter 2011; Slootman and Tillie 2006; Van San et al. 2010, 2013).

Lobermeier (2006) observed the same uncertainty in parents who were confronted with their child’s radicalization into right-wing extremism. His empirical study on right-wing extremism and upbringing has shown that, usually, parents initially try to talk reason into their children; however, when reason fails, many parents feel that it is no use to respond to their child’s ideology and behavior. In favor of a good relationship, parents then decide to ignore it (Lobermeier 2006). Other reactions found by Lobermeier (2006) were acceptance, tolerance, and prohibition of interference with extreme right-wing ideology. Parents’ responses were determined by their desire to stay in touch with or reconnect with their son or daughter.

The above-mentioned studies were based on small field samples, and the studies of Lobermeier (2006) and Becker (2008) solely focused on young people with right-wing ideals. This article is based on a much larger field study than the previous research: 82 in-depth interviews were held in Belgium and the Netherlands with adolescents and young adults who hold (extreme) ideals. Interviews were also held with the parents or siblings of each adolescent and young adult. Furthermore, as distinct from the research of Lobermeier (2006) and Becker (2008), this research included young people from various groups: right-wing extremists; Muslim radicals; and ultra-left-wing groups, such as animal rights activists, anarchists, and antifascists. We included such different groups, because research shows that the radicalization process of these adherents of widely divergent ideologies occurs in similar ways (Gielen 2008; Stern 2003). Our study focuses on the family dynamics within the radicalization process and explores how parents react to the development of extreme ideals. The article will try to answer the questions: How do parents respond towards the (extreme) ideals of their children, and why do they respond in this way? It is important to explore the parental reaction, as the influence of the parent on the development of the child is crucial; however, it is not exclusive (Borkowski et al. 2009; Bronfenbrenner 1986; Guajardo et al. 2009; Meadows 1996).

## Method

### Participants

#### Adolescents and young adults

Forty interviewees were still involved with radical groups, and eight interviewees were former radicals. In this research, we define a former radical as someone who once had extremist ideas or carried out extremist behavior but has been de-radicalized or has disengaged from radical groups. Neumann (2010) defined *de-radicalization* as a substantive change in ideology. *Disengagement* facilitates behavioral change, such as rejection of violence (Horgan and Braddock 2010). It follows that disengagement does not require a change in the radical ideas as such, although it does require renouncement of violence as a method of striving for change. As for the former radicals we interviewed, they have been de-radicalized from 1 to 9 years.

The age of our young respondents ranged from 16 to 33 years, with a mean of 22.06 years. Their ideological search commenced between 10 and 19 years old, and radicalization took place at an average age of 16.7 years. People were eligible for inclusion in this study if they (had) pursued ideals that harmed the democratic rights of others, used or condoned violence in pursuit of their ideals, or if they or their parents indicated that they (had) held radical beliefs.

Twenty-six out of the forty-eight young respondents held, or previously held, extreme Islamic beliefs; sixteen of them are converts. Thirteen respondents sympathize(d) with extreme right-wing or national socialist ideologies. Six respondents are, or were, involved in violent animal rights activism, and three respondents supported extreme left-wing ideologies, like anarchism and antifascism. Twenty-nine of the young respondents were men, and nineteen were women (see also Table 1).

**Table 1:** Number of Interviewees and their (Child’s/Siblings’) Ideologies

Ideology	Total—young respondents	Involved in ideology at time of interview	Former radicals	Male	Female	Parents	Siblings
Extreme Islam	26	24	2	13	13	14	3
Extreme right-wing	13	8	5	12	1	8	3
Animal rights	6	5	1	2	4	4	0
Extreme left-wing	3	3	0	2	1	2	0
Total	48	40	8	29	19	28	6

#### Family members

Twenty-eight parents and six siblings were interviewed. By including parents and siblings in the research, we aimed for triangulation. We spoke with eighteen mothers, eight fathers, and two stepfathers. Six parents were still married to the other parent, twenty parents were divorced, and two mothers were single mothers. The siblings were all sisters: two younger and four older.

### Procedure

The research took place in Belgium and the Netherlands. The fieldwork was conducted between January 2012 and May 2015, in a time that polarization was increasing in both societies, but before the terror attacks that took place later that year in Belgium. The majority of our respondents were recruited on Facebook, via the creation of a neutral researcher Facebook account. On our profile we explicated our research and goals. Next, we searched Facebook for adolescents and young adults (between 15 and 30 years of age) who explicitly displayed their ideals on their profile pages. We approached potential respondents if their profiles, for example, disclosed admiration of martyrs or white supremacists, or displayed anti-government statements. We also joined ideological groups on Facebook and approached active members for an interview.

Via a private Facebook message, we asked potential respondents to participate in an interview about their ideals. In this message, we introduced and clarified the research. Because Facebook has ownership over all sent messages, we did not conduct interviews online; instead, we invited people for a face-to-face interview. We interviewed our respondents offline so that no one would have ownership of the interview data, which could potentially harm our interviewees. All our respondents gave verbal consent to participate in our research and to audio record the interview. We also received verbal parental consent for the included participants who were between 16 and 18 years old.

For this study, in-depth interviews were conducted, using prepared topic lists. To obtain information about parental reactions, we asked young people and their parents how the father, mother, or both reacted when the child became involved with extreme ideals. They were also asked about the parental sentiments towards the ideals and whether any boundaries were set regarding the pursuit of the ideals. The face-to-face interviews were held at locations favored by the respondents, for example, in local shopping malls, public libraries, on park benches, or at their homes. We audio recorded the interviews and then made a verbatim transcription. All information that could lead to our participants was deleted and pseudonyms were used, in order to guarantee anonymity. The research received ethical approval from the Faculty Ethics Review Committee of Utrecht University in the Netherlands.

#### Interviews with family members

In our pilot study (Van San et al. 2010), we found that parents of young people with extreme ideals were difficult to find; therefore, most parents were approached through their children. We found that we were often able to speak with the parents in cases where the child and his or her parents shared their ideological views. On the other hand, in cases where the parents and the child disagreed on the ideals, this often meant that the parent and child had a difficult relationship, and the young respondent would then forbid us from contacting the parent. As it was sometimes difficult to gain consent from the young respondents to contact their parents, we interviewed siblings as well. We asked the siblings about the home situation, why their brother or sister radicalized, and how their parents reacted upon the radicalization. Furthermore, we were able to speak to some parents whose child had left the country to fight for his or her ideals in Syria. In those cases, we did not speak to the child, but the parents were able to teach us more about the pedagogical context of their child’s radicalization.

### Data Analyses

We used NVivo10 software to analyse our interview data. To obtain researcher triangulation, two researchers conducted the analysis. We started our open coding by labeling four interviews. One researcher began the analysis by openly coding four interviews with adolescents and their parents. What helped us to focus were the themes and topics we asked about during the interviews. The second researcher tried to code the interviews using the same labels, resulting in a more reliable list of open codes. One of the most apparent labels was the reaction of parents to their children’s ideology. Axial coding was accomplished for further analysis of the different kinds of reactions.

Analyst triangulation was obtained by peer debriefing: a research group that consisted of five co-researchers provided the authors with feedback on their analysis. Furthermore, inter-rater reliability was obtained through the repeated coding of the interviews by another researcher (kappa was 0.93 after disagreements in coding were discussed and consensus was reached), and through individual classification of the parental reactions by the two researchers.

## Results

Differences in parental reaction emerged from the accounts of adolescents and their parents. Four parental reactions to radicalization could be discerned: reject, ignore, applaud, and discuss. Parents who *rejected* their child’s extremist ideals were unsupportive of his or her ideological position and tried to control it. Parents who *ignored* their child’s ideology did not support their child in his or her beliefs nor did they impose limits on their child’s behavior. Parents *applaud* in cases where the parent supported the child’s extreme ideas and did not enforce any limits. Parents’ reactions were scored as *discuss* when parents reacted in a supportive yet controlling way. These four reactions are illustrated below.

### Reject

Many parents responded with rejection towards their children’s ideals: they were unsupportive of the ideals and, moreover, tried to control the ideals. These rejective reactions of parents were coded into three different types: (1) a rejective reaction in which the parent disagreed with the child; (2) a rejective reaction in which the parent would forbid the ideals; and (3) a rejective reaction that was led by incomprehension (see Table 2).

**Table 2:** Different types of parental reactions towards extreme ideals as found in the Ideals Adrift II study

Type of reaction	Number of interviewees that mentioned this reaction	Number of times that the reaction was mentioned
Reject	45	115
Disagreement	39	64
Forbid	15	25
Incomprehension	22	27
Ignore	51	162
Disassociate	14	21
Own choice	20	30
It will pass	17	22
Powerlessness	18	41
Reaction or ban does not help	17	23
Other	20	33
Applaud	37	55
Discuss	23	45

A reaction was scored as a “disagreement” if the parent explicitly disagreed with or part of their child’s ideology. If, for example, the adolescent notes that his or her ideals had led to an enormous dispute with his parents, this was scored as parents who disagree. Or, if, for example, parents proclaimed that they detested their child’s ideology or that they hated the way their child was dressed, this was coded as a disagreement. Some parents disagreed with the ideal in itself; other parents were fine with the ideals but thought that the way the child declared his or her beliefs was too extreme. These parents disagreed with the extent that their children wished to live up to their ideals, and they responded by rejecting the intensity. The parents of the converted girls, Samira (18 years old) and Sophie (19 years old), for example, had no problems with the fact that their daughters became religious, but they felt that their daughters overacted.

She found it hard to tell me, because she also wanted to wear a headscarf. Well… I needed a minute to digest that [laughs]. I thought, ‘If you really want that… a nice little headscarf isn’t that bad’. But it has become more and more strict because the curtains in the house should now be closed when she walks around without a [headscarf]. And no, we don’t want that, so she wears a headscarf when she’s here, because it’s possible that a man looks into the house [laughs]. It’s all very extreme. (Mother of Sophie)

This did not solely apply to converts. The mother of Khadija is Muslim, but she disagreed with the way her daughter put her beliefs into practice. Khadija (18 years old) preferred to wear a dark colored *khimar* and *jellaba* (i.e., long clothing); whereas, her mother believed a colorful headscarf would be sufficient. In some cases, the fights became so intense that they caused a break-up between parent and child. This was especially the case when parents forbade the ideals. For example, Jelmer (28 years old), who was banned from his mother’s house after converting to Islam said,

I told my mum: well, I became Muslim. My mum then was like: no, you can’t be serious. She was really angry. We got into a fight and she kicked me out. She also said: you’re no longer my son… Well, so be it.

A rejective reaction was scored, as caused by incomprehension, if the parent or the child stated that the parent was unhappy about the ideals, because they did not understand the ideals or did not have any substantive knowledge about the ideals. Yusuf (23 years old), for example, converted to Islam and felt that his parents did not understand his new beliefs:

I often talk to my parents about these matters, but Allah said that non-believers have a hijaab [veil] in front of their eyes. So, even if they want to understand it, they cannot. Even if you rub someone’s nose in it, they would still not see it. Why? Because their heart is closed to Islam.

### Ignore

In our interviews, we found that many parents reacted by “ignoring” the ideals of the child: these parents did not support the ideals of the child nor did they put up boundaries. We distinguished six different kinds of ignoring reactions (see Table 2). These reactions do not mean that a parent just neglects the child and his or her ideology, but, for example, they imply that parents think it might simply be a phase that will pass. Another ignoring response was that parents considered it to be their child’s own choice, and so they did not interfere. Other parents disassociated from the child, because they did not want or know how to handle the ideology of the child.

From our study it appeared that parents sometimes disassociated themselves from the child and his or her ideas, because they struggled with problems of their own (e.g., alcoholism, loss of partner, depression, divorce, etc.) and were unable to devote attention to understanding the ideals of their child. In at least 18 families, there were severe problems with alcohol, drugs, sickness or depression. Some were caused by divorce or by the death of a family member. Twenty-eight of the fifty young respondents we interviewed had lost a parent due to divorce or death. Bas (22 years old), an animal activist, told us that his mother did not know everything he had been up to, because after his father died, “the parenting stopped”. “Since puberty, I wasn’t really [being] parented anymore, because everyone was mourning of course.” The mother of Nabil (18 years old), a young Muslim who left to fight in Syria, also pointed out that, due to several problems, there was not a lot of reaction at home: “His father is very depressed, and he is also physically ill, so he sleeps a lot. He has to take his medicines four times a day. So, at the age of 16, he [Nabil] was basically all on his own.”

Other parents chose to ignore the ideals more intentionally. Evelyn (19 years old), for example, is a small-town girl who converted to Islam when she was 17 years old. Her father was not fond of her being Muslim; and apart from setting some limits, he reacted by ignoring the ideals, because he believes it is his daughters’ own choice. He explained,

You get used to it. Except for that burka, that was the limit, but other than that she can do whatever she wants. If only she doesn’t bother me with it. She shouldn’t say things like, ‘dad, Allah does not allow you to do that’, because then I would say, ‘there’s the door from which you can leave’. That’s not how we’re going to do it here. You don’t interfere with my life and I’ll stay out of yours.

We saw similar ignoring reactions in other parents: “it’s [his or her] life; they should see it for themselves”, was a very common reaction amongst parents. Furthermore, there were parents who did not react, because they believed that it was just a phase, something that would subside after late adolescence. As Albert (18 years old), a national socialist, said of his parents: “back then they didn’t mind so much. They thought it was a phase.”

Another ignoring reaction seemed to be caused by a sense of powerlessness: parents did not have “the tools” to respond to the radicalization of the child. Both Sophie’s father and Sylvia’s (22 years old) mother did not know what to look out for; thus, they stood on the sidelines:

I should have supported her more, like ‘to which mosque are you actually going?’ But I didn’t know shit about it myself either. There are many moderate mosques, but also many rigid mosques, and she ended up with one of these Sunni mosques. But if she, for example, went to one of those moderate mosques, she would have kept much more connection with Western society. (Father of Sophie).

I was tackled about it at school as well, because at school they thought it was abnormal. And I said, “well yes, what am I supposed to do? What am I supposed to think about her rolling up her jeans and wearing those army boots?” See, if you don’t know! Because at that time I wasn’t even aware of that. (Mother of Sylvia).

Furthermore, there were parents who considered that responding to, or prohibiting, certain ideas would be useless. According to the mother of Sophie, a girl who converted to Islam, it would be no use to try to forbid her child from engaging with certain ideals. She felt that her daughter would go along with it anyway and would leave home if she prohibited her ideals and would lose contact. The mother of Thijs (18 years old) also felt that she could not stop her son from proclaiming his right-wing ideals, as he would go on doing it anyway, and there would be no way to prevent that. “So now I just think, ‘okay Thijs, if that’s your idea, then fine’. I don’t feel like stressing about it anymore”. The mother of Dylan (16 years old) felt the same way. She let her son say whatever he wanted. “The more you ignore him, the less he talks about [National Socialism]. Because if he knows he is getting to you, he will talk like that on purpose.”

### Applaud

In many interviews an applauding reaction was mentioned (see Table 2). Parental reactions were scored as applauding in cases where the parent or child indicated that the parent supported the ideals and did not put up any boundaries. Parents responded in an applauding manner towards their children’s choice of religion, because they supported the idea of their children searching for meaning in life. They found it positive that the child was trying to develop his or her spiritually. The mother of the converted Leonie (17 years old) said in her interview:

What I especially like about it is that she’s looking for deeper understanding and, apparently, doesn’t want to live a superficial life. That’s what I like about it. She’s not just after money or aiming for a career.

Other parents reacted with applause towards their children’s ideology, because they admired their ideas, or because they shared their ideas. When young people from Muslim families, for example, decided to practice the Islamic religion, they usually found no resistance. Most Muslim parents supported their children in their search for Islam and let them develop freely, as is demonstrated in the interview with Hossain (20 years old): “They don’t think… they think it’s good, right. They are Muslims too, it makes them happy.”

It was only after the ideals became extreme, that the parents changed their initial applauding in rejection. The mother of Tarik (25 years old) recalls how she liked the religious involvement of her son at first as she did not know that Shariah4Belgium was a radical Salafist organization:

It was not until Tarik left [to travel to Syria] that I noticed what a negative influence they [Shariah4Belgium] had on young people. But at the time that this organization came to exist, I didn’t gk badly about them.

### Discuss

There were several parents in our study that reacted in a supportive and controlling way towards the development of ideals in their children. These parents stated that they handled the new situation by discussing the ideals with their child; by monitoring their child’s whereabouts or thought patterns through regular communication; or by attending ideology-related gatherings with him or her (see Table 2). The mother of Leonie (17 years old), for example, joined her daughter in visits to the mosque to show interest in her daughter’s religion and to find out what was preached. The mother of Patrick (17 years old) tried to keep a finger on the pulse as well:

Interviewer: But as a mother, how do you make sure that such a radical statement does not change into action? Mother: Well… how do you make sure? You cannot really. You can try to talk about it at the very most, and check regularly, ‘what about now? How do you feel about it now?’ So, by those means you can keep your finger on the pulse.

## Discussion

This study found that parents respond in different ways toward extreme ideals in their children: by rejecting, applauding, discussing, or ignoring the ideals. The study shows that many parents did not support the ideals of their child nor put up any boundaries. Parents who responded without support or control seemed to have been led by a sense of powerlessness: they did not have “the tools” to respond to the radicalization of the child. Similar to the previous research of Kerr et al. (2009), we found that parents often disengage when their children begin to radicalize. Most parents in our research struggled to cope with radicalization and were unsure of how to respond: they were unaware that some ideals need a parental reaction, instead of simply assuming this is a phase in adolescence. Other parents felt powerless and did not know how to respond. A parental uncertainty exists within these parents, and parents do not know whom to turn to for support. In our pilot study (Van San et al. 2010) we found the same lack of responsiveness: most parents did not respond to or intervene in the radical behavior of the child. The dominant reaction in parents was from a relativistic approach to parenting, as the parent considered the ideals to be the child’s own choice. Becker (2008) and Lobermeier (2006) came to the same conclusion. The findings of this systematic qualitative research are in keeping with the previous research and reinforce their findings.

### Strengths and Limitations

An important strength of this study was the establishment of categories for parents’ reactions toward radicalization in a Western European context. The study was able to show that multiple reactions toward radicalization exist, and parents have different reasons for responding the way they do. However, when considering the categories of parental reactions, the selection bias in the parents we interviewed has to be taken into account. It was often challenging to gain consent from the young respondents to speak to their parents and, therefore, we came across certain difficulties. In cases where the respondent and his or her parents agreed on the ideology that he or she strived for, we were often able to speak to the parents. However, in cases where the parents and the child disagreed on the ideals, this often meant that they had a difficult relationship, which made it impossible for us to contact the parent. In these cases, we only know how the parents responded and socialized from the perspective of the young respondent.

A further important point that needs to be addressed is that parenting is, of course, never the only factor that influences the radicalization process. Feelings of relative deprivation, powerlessness, the influence of peers, the use of the internet, and even, simply, maturation, are said to relate to radicalism as well (Adelson 1975; Benschop 2006; Buijs et al. 2006; Helmus 2009; Pels and De Ruyter 2012). Individuals can also feel attracted to certain ideologies in their search for excitement (i.e., novelty-seeking) or in their search for belonging, because a shared ideological commitment is a typical group activity (Crenshaw 2000; Fermin 2009; Noppe et al. 2010; Van der Pligt and Koomen 2009). Parental reactions towards radicalization, on the other hand, are hardly ever mentioned but should be considered as important possible influences.

### Future Directions

A few of the parents involved in our research reacted in a supportive yet controlling way. We tentatively suggest that their children were developing strong ideals, but they kept within the law. It would be interesting to explore whether support and control do indeed have a de-radicalizing effect. As previous research showed that parental warmth, combined with control, produces the best child outcomes in terms of health and social development (Maccoby and Martin 1983). Parents who want to prevent radicalization should not react simply by forbidding extreme behavior, as a democratic society asks more of its citizens (De Winter 2016). Establishing and enforcing limits should be part of the response, but it is more important to teach young people that there are non-violent ways to change society and get one’s voice heard. Young people’s energy and willingness to change the world should be tempered by instruction in how to achieve goals by arguing, lobbying, and organizing; thus, by channeling their youthful energy and willpower constructively (Davies 2016).

Our research confirmed, however, that parents feel uncertain about how to react when their children move toward extreme ideologies and would like to have tools to prevent radicalization. The interviews, for example, showed that parents often had no knowledge about the religious or political views that their child is developing; therefore, it was hard for them to discuss these ideals or set boundaries when needed. Moreover, when their children go through a transition in moving from adolescence to adulthood, parents seem to struggle to guide them through this transition. Parents need information about different kinds of extreme ideology, the process of radicalization, how to respond to radicalization, and how to guide their children’s identity development and transition to adulthood (Sikkens et al. 2017). Also, the forces that influence young people are often too big and complex for parents to counter alone. Earlier research showed that when young people are at risk of radicalization, it is helpful to provide parents with professional support for helping them to discuss extreme ideas with their children and offer alternative perspectives (Gielen 2015). Future research could explore how professionals could support parents to enable them to react constructively when children show an interest in extreme political and religious ideals, help them discuss complicated political issues and existential questions, and guide them through the child’s transition from adolescence to adulthood.
